# Gender and post-ischemic recovery of hypertrophied rat hearts

**DOI:** 10.1186/1471-2261-6-8

**Published:** 2006-03-01

**Authors:** Ramesh Saeedi, Richard B Wambolt, Hannah Parsons, Christine Antler, Hon S Leong, Angelica Keller, George A Dunaway, Kirill M Popov, Michael F Allard

**Affiliations:** 1 James Hogg iCAPTURE Centre for Cardiovascular and Pulmonary Research, Department of Pathology and Laboratory Medicine, University of British Columbia-St Paul's Hospital, Vancouver, BC, V6Z 1Y6, Canada; 2 Labatoire CRRET, Faculté des Sciences, Université de Paris XII, Creteil Cedex, 94010, France; 3 Department of Pharmacology, Southern Illinois University School of Medicine, Springfield, IL, 62794, USA; 4 Department of Biochemistry and Molecular Genetics, University of Alabama at Birmingham, Birmingham, AL 35294, USA

## Abstract

**Background:**

Gender influences the cardiac response to prolonged increases in workload, with differences at structural, functional, and molecular levels. However, it is unknown if post-ischemic function or metabolism of female hypertrophied hearts differ from male hypertrophied hearts. Thus, we tested the hypothesis that gender influences post-ischemic function of pressure-overload hypertrophied hearts and determined if the effect of gender on post-ischemic outcome could be explained by differences in metabolism, especially the catabolic fate of glucose.

**Methods:**

Function and metabolism of isolated working hearts from sham-operated and aortic-constricted male and female Sprague-Dawley rats before and after 20 min of no-flow ischemia (N = 17 to 27 per group) were compared. Parallel series of hearts were perfused with Krebs-Henseleit solution containing 5.5 mM [5-^3^H/U-^14^C]-glucose, 1.2 mM [1-^14^C]-palmitate, 0.5 mM [U-^14^C]-lactate, and 100 mU/L insulin to measure glycolysis and glucose oxidation in one series and oxidation of palmitate and lactate in the second. Statistical analysis was performed using two-way analysis of variance. The sequential rejective Bonferroni procedure was used to correct for multiple comparisons and tests.

**Results:**

Female gender negatively influenced post-ischemic function of non-hypertrophied hearts, but did not significantly influence function of hypertrophied hearts after ischemia such that mass-corrected hypertrophied heart function did not differ between genders. Before ischemia, glycolysis was accelerated in hypertrophied hearts, but to a greater extent in males, and did not differ between male and female non-hypertrophied hearts. Glycolysis fell in all groups after ischemia, except in non-hypertrophied female hearts, with the reduction in glycolysis after ischemia being greatest in males. Post-ischemic glycolytic rates were, therefore, similarly accelerated in hypertrophied male and female hearts and higher in female than male non-hypertrophied hearts. Glucose oxidation was lower in female than male hearts and was unaffected by hypertrophy or ischemia. Consequently, non-oxidative catabolism of glucose after ischemia was lowest in male non-hypertrophied hearts and comparably elevated in hypertrophied hearts of both sexes. These differences in non-oxidative glucose catabolism were inversely related to post-ischemic functional recovery.

**Conclusion:**

Gender does not significantly influence post-ischemic function of hypertrophied hearts, even though female sex is detrimental to post-ischemic function in non-hypertrophied hearts. Differences in glucose catabolism may contribute to hypertrophy-induced and gender-related differences in post-ischemic function.

## Background

There is substantial clinical and experimental evidence to indicate that gender influences the myocardial response to hemodynamic overload with female sex having a beneficial effect [1-5]. In the clinical setting, for example, studies have reported that females have increased cardiac hypertrophy, more concentric remodelling, and better preservation of left ventricular systolic function than males in response to increased hemodynamic load, such as occurs with aortic stenosis or hypertension [4,5]. Experimental studies have also found differences in geometric remodelling and cardiac function between hypertrophied hearts from males and females similar to those observed in humans [1-3]. This gender-related preservation of function in the setting of cardiac hypertrophy, which is a leading risk factor for development of heart failure [6], translates into a lower overall incidence of heart failure in females than in males [7].

Despite having a beneficial effect on cardiac remodelling due to hemodynamic overload, female sex is associated with greater rates of heart failure in the presence of symptomatic coronary artery disease [7,8]. This finding is consistent with clinical data indicating that, although of lower incidence in women, coronary artery disease is particularly problematic once present. Correspondingly, females have an increased risk of a poor outcome after coronary revascularization procedures [7,9,10] and younger women have worse short-term outcomes after acute myocardial ischemia leading to infarction than men [11,12]. Furthermore, the presence of left ventricular hypertrophy has been shown to have a detrimental impact on mortality due to ischemic heart disease that is greater in women than in men [13]. Taken together, these data indicate that female sex influences remodelling due to hemodynamic overload as well as outcome after ischemia. They also suggest that the functional significance of simultaneously occurring cardiac hypertrophy and coronary artery disease differs between women than in men.

Cardiac hypertrophy due to hemodynamic overload has generally been viewed as an adaptive response [14,15]. However, it can also be considered maladaptive. This latter consideration becomes especially apparent when hypertrophied hearts are exposed to ischemia and reperfusion, a situation that results in significantly lower post-ischemic heart function in hypertrophied hearts than in non-hypertrophied hearts [16-19]. Currently, the detrimental effect of hypertrophy on post-ischemic heart function is well documented in hypertrophied hearts from males. To our knowledge, functional outcome of pressure-overload hypertrophied hearts after ischemia in females and whether cardiac hypertrophy is more or less detrimental to outcome in females than in males are not yet known with certainty. Such information would provide important insights into the apparent additive effects of cardiac hypertrophy and ischemic heart disease in females observed clinically.

As in non-hypertrophied hearts [20], the catabolic fate of exogenous glucose is recognized as an important factor that contributes to the poor post-ischemic function of hypertrophied hearts, at least in hypertrophied hearts from males [16-19,21,22]. Specifically, the extent to which glucose passing through glycolysis is catabolized non-oxidatively (i.e, is converted to lactate rather to CO_2_) appears to be of functional significance, as rates of non-oxidative glycolysis are inversely related to post-ischemic function of male hypertrophied and non-hypertrophied hearts [22]. An acceleration of overall glycolysis combined with a limitation of glucose oxidation [19,23,24] result in increased rates of non-oxidative glycolysis in hypertrophied male hearts [22]. That accelerated rates of non-oxidative glycolysis contribute to the poor outcome of hypertrophied hearts after ischemia is supported by data showing that stimulation of glucose oxidation and/or reduction of glycolysis, effects that alone or in combination reduce non-oxidative glycolysis, substantially improve function of ischemic-reperfused hypertrophied hearts from male rats [19,22]. At this time, it is not yet clearly known if female gender influences the pattern of substrate use and particularly of glucose use in pressure-overload hypertrophied hearts either before or after ischemia.

In the experiments described here, we tested the hypothesis that gender influences post-ischemic functional recovery of pressure-overload hypertrophied hearts. Given the importance of substrate use to post-ischemic recovery of hypertrophied heart function in males, we also determined if substrate use in female hypertrophied hearts differed from that in male hypertrophied hearts and, if so, whether any differences could be explained by alteration in expression or activity of key enzymes and proteins involved in control of myocardial substrate use.

## Methods

### Animal model

A mild pressure-overload left ventricular hypertrophy was produced in male and female Sprague-Dawley rats by constriction of the suprarenal abdominal aorta with a metallic clip (0.4 and 0.3 mm diameter, respectively) at 3 weeks of age [23]. In sham-operated control rats, the aorta was isolated, but not clipped. Experiments were performed 8 weeks after surgery. Food and water were administrated ad libitum. These experiments were approved by the institutional committee on the use of laboratory animals in research and conform with the *Guide for the Care and Use of Laboratory Animals *by the US National Institutes of Health (NIH Publication No. 85-23, revised 1986).

A subset of anesthetized (ketamine/xylazine, 90/10 mg/kg, IP) male and female rats with and without aortic constriction were studied 8 weeks after surgery in order to determine if gender influenced the pressure load in this model. Mean arterial pressure and heart rate were determined by means of a micromanometer-tipped catheter (SPR-838, Millar Instruments, Houston, TX) in the left carotid artery.

### Isolated heart preparation and perfusion protocol

As previously described [19,23], isolated working hearts from aortic-constricted and sham-operated rats were perfused at a left atrial preload of 11.5 mmHg and an aortic afterload of 80 mmHg in a closed recirculating system with oxygenated (95% O_2_-5% CO_2_) Krebs-Henseleit (KH) solution. Hearts were all perfused under comparable conditions in order to avoid potentially confounding effects of exposure to different afterloads. Although hearts from aortic-constricted rats are exposed to elevated afterloads in vivo, we have shown that adjusting afterload to normalize coronary flow per gram in isolated working hypertrophied hearts does not significantly influence the functional or metabolic outcomes observed after ischemia [25]. The KH solution contained 1.2 mM palmitate prebound to fatty acid-free albumin (3%), 5.5 mM glucose, 0.5 mM lactate, 2.5 mM calcium, and 100 mU/l insulin and was maintained at 37°C. A high physiologic concentration of insulin was used in order to ensure that insulin-dependent glucose uptake was not limiting. The concentration of palmitate was chosen in order to recapitulate conditions observed during reperfusion after ischemia [20]. Heart rate, peak systolic pressure, cardiac output and aortic flow were measured every 10 min of the working heart perfusion. Coronary flow and indices of external cardiac work, including rate-pressure product and hydraulic power, were calculated as described [19,23].

Hearts were initially perfused for 30 min under normoxic non-ischemic conditions followed by a 20 min period of global ischemia that was induced by clamping both the left atrial preload and aortic afterload lines. At the end of ischemia, the clamps were removed and the hearts were reperfused for 40 min. We have previously shown that function is stable in isolated working hearts perfused under non-ischemic, normoxic conditions for 90 min [26]. At the end of reperfusion, hearts were quickly frozen using tongs cooled to the temperature of liquid nitrogen. Frozen heart tissue was weighed with a portion of ventricular tissue used to determine the dry-to-wet tissue weight ratio.

### Measurement of myocardial substrate utilization and glycogen content

Myocardial substrate utilization was measured in two parallel series in each group. In the first series of experiments, hearts were perfused with [5-^3^H]-glucose and [U-^14^C]-glucose in order to determine rates of glycolysis and glucose oxidation, respectively, as previously described [23,27]. In the second series of experiments performed under identical conditions, rates of lactate and palmitate oxidation were determined by perfusing the hearts with [U-^14^C]-lactate and [9,10-^3^H] palmitate [23,27]. It should be noted that these rates refer to catabolic rates of exogenous substrates, as the contribution of endogenous substrates, such as glycogen, was not taken into account. Rates of glycolysis and palmitate oxidation were determined by quantitatively measuring the rate of ^3^H_2_O production. Rates of glucose and lactate oxidation were measured by quantitative collection of ^14^CO_2 _released as a gas and dissolved in the perfusate as [^14^C]-bicarbonate. Perfusate and gaseous samples were taken every 10 min of perfusion and were ultimately placed in vials containing scintillation cocktail and counted by standard techniques. Pre-ischemic values for all metabolic rates were calculated based on data collected between 10 and 30 min, while post-ischemic values were calculated between 10 and 40 min of reperfusion. Rates of non-oxidative glycolysis were calculated as the difference between rates of glycolysis and glucose oxidation [22]. Myocardial glycogen was determined by measuring glucose obtained following digestion of frozen powdered ventricular tissue with 30% KOH, ethanol precipitation, and acid hydrolysis of glycogen [28].

### Content and activity of selected myocardial metabolic proteins and pnzymes

The content of selected enzymes and proteins involved in control of myocardial substrate utilization was determined by immunoblot analysis using a previously described method [26,29]. The particular proteins and enzymes assessed were chosen based upon either recognition as having significant control strength for the metabolic pathway in question and/or reported alteration in models of cardiac hypertrophy. Briefly, samples of frozen ventricular tissue homogenate (containing 20 μg total protein) were solubilized by boiling in reducing sample buffer, separated by electrophoresis on 10% SDS-polyacrylamide gels, and transferred by electroblotting to a nitrocellulose membrane. After non-specific blocking, the blots were probed overnight with the following primary antibodies: rabbit anti-GLUT-4 (1:1500 dilution, Cell Signalling Technology, Missisauga, Ontario), mouse anti-glyceraldehyde-3-phosphate dehydrogenase (GAPDH, 1:200,000 dilution, Molecular Probes, Eugene, Oregon), rabbit anti-α- and β-enolase (1:5,000 and 1:75,000 dilution, respectively [30], rabbit anti-muscle type phosphofructokinase (PFK, 1:3,000 dilution [31], rabbit anti-pyruvate dehydrogenase complex (PDC, 1:4,000 dilution, [32], rabbit anti-medium-chain (MCAD, 1:4,000 dilution) and anti-long-chain (LCAD, 1:5,000 dilution) acyl-CoA dehydrogenases [33]. After incubation with the appropriate secondary antibody, the signal was detected by an ECL based detection system. Band intensity values for each individual protein were quantified by densitometry. For each protein of interest, GAPDH was sequentially detected for purposes of normalization. Background-corrected densitometry values for the protein of interest and GAPDH were used to calculate relative expression ratios. Activity of PDC was determined as described [32].

### Statistical analysis

Analysis was performed using SPSS version 7.0. Weight, glycogen, expression, and activity data were analyzed using two-way analysis of variance (ANOVA). Left ventricular function, glycolysis, and oxidation of glucose, lactate, and palmitate were examined using two-way repeated measures ANOVA. A log transformation of data was used, when necessary, to satisfy homogeneity of variance assumption for ANOVA. The sequential rejective Bonferroni procedure was used to correct for multiple comparisons and tests. A corrected p value > 0.05 was considered as non-significant. Data are expressed as means value ± SEM.

## Results

### Animal model

Morphologic and in vivo hemodynamic data are summarized in Table 1. Eight weeks after surgery, heart weight in male and female aortic constricted rats was approximately 20% greater than sex-matched sham-operated control rats. There was no significant difference in body weight between aortic-constricted and sham-operated rats of either sex. Heart and body weights of female rats were significantly lower than that of male rats. As with heart weight, the ratio of heart weight and body weight showed a comparable percent increase in both male (20.1 ± 2.3%) and female (24.0 ± 1.9%, p = NS) aortic-constricted rats. Aortic constriction resulted in significant increases in mean aortic pressure in both male and female rats but had no effect on heart rate. Mean aortic pressure did not differ between male and female sham-operated control rats or between male and female rats with aortic constriction.

**Table 1:** Morphologic and hemodynamic data of hearts from sham-operated and aortic-constricted rat hearts

	Male		Female	
		
	Control	Hypertrophy	Control	Hypertrophy
Body wt (g)	451 ± 5	453 ± 10	278 ± 5$	271 ± 5*$
Heart wt (g)	1.86 ± 0.02	2.23 ± 0.04*	1.28 ± 0.04$	1.55 ± 0.03*$
Heart/body wt	4.1 ± 0.04	4.95 ± 0.09*	4.61 ± 0.11$	5.72 ± 0.09*$
Heart rate (bpm)	262.5 ± 7.4	255.8 ± 3.0*	260.4 ± 10.1	255.4 ± 10.5*
Mean aortic pressure (mm Hg)	109.6 ± 7.0	164.6 ± 7.3	96.3 ± 6.0	153.1 ± 5.8

Values are Mean ± SEM. Sham-operated rats, Control. Aortic-constricted rats, Hypertrophy. Numbers for morphologic data are 18–36 per group and for hemodynamic data are 6–8 per group; * vs. Sex-matched Control, p < 0.05; $vs. corresponding Male value, p < 0.05.

### Heart function

Heart function is summarized in Table 2. Before ischemia, cardiac output, hydraulic power, and coronary flow were lower in male hypertrophied hearts than corresponding values in male non-hypertrophied hearts. In contrast, only coronary flow was lower in hypertrophied hearts from female rats than in non-hypertrophied hearts from female rats. Other than higher coronary flow rates in females, there were no significant differences in function between male and female hypertrophied hearts. In non-hypertrophied hearts, cardiac output and hydraulic power were higher in males than in females, while coronary flow was highest in females.

**Table 2:** Heart function data in hearts from sham-operated and aortic-constricted rat hearts

	Male Control		Male Hypertrophy		Female Control		Female Hypertrophy	
				
	Pre-I	Post-I	Pre-I	Post-I	Pre-I	Post-I	Pre-I	Post-I
Heart Rate (bpm)	253 ± 4	261 ± 5	247 ± 5	242 ± 10	250 ± 3	214 ± 7#$	251 ± 5	182 ± 11#*$
Peak Systolic Pressure (mmHg)	111 ± 1	100 ± 2#	109 ± 2	95 ± 2#	108 ± 1	91 ± 4#	108 ± 1	89 ± 4#$
Cardiac Output (ml/min)	69 ± 2	47 ± 3#	56 ± 2*	31 ± 4#*	55 ± 1$	25 ± 2#$	53 ± 2	20 ± 2#$
Coronary Flow (ml/min g wet wt)	16 ± 1	13 ± 1#	10 ± 1*	6 ± 1#*	20 ± 1$	11 ± 1#$	14 ± 1*$	6 ± 1#*
Rate Pressure Product (bpm × mmHg × 10^-3^)	28 ± 1	26 ± 1#	27 ± 1	24 ± 1#*	27 ± 1	20 ± 1#$	27 ± 1	17 ± 1#$
Hydraulic Power (ml/min × mmHg × 10^-3^)	77 ± 3	48 ± 4#	62 ± 3*	30 ± 4#*	59 ± 2$	24 ± 2#$	57 ± 2	19 ± 2#*$

Values are Mean ± SEM. Non-hypertrophied heart, Control. Hypertrophied heart, Hypertrophy. Pre-I, Pre-ischemia; Post-I, Post-ischemia; *, vs Sex-matched Control; #, vs pre-Ischemic values; $, vs corresponding Male value; all at p < 0.05.

**Figure 1:**
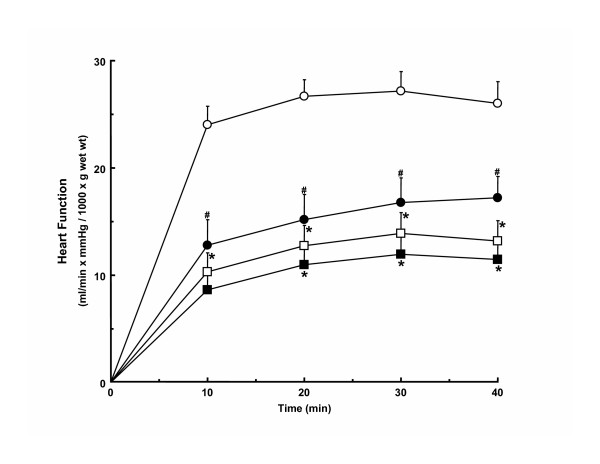
Post-ischemic mass-corrected function of hypertrophied and non-hypertrophied hearts from male and female rats. Open circle, male non-hypertrophied hearts; open square, male hypertrophied hearts; filled circle, female non-hypertrophied hearts; filled square, female hypertrophied hearts. *, vs. sex-matched non-hypertrophied hearts (p < 0.05). #, vs. corresponding male hearts (p < 0.05). Numbers per group: 27 male non-hypertrophied hearts, 21 male hypertrophied hearts, 17 female non-hypertrophied hearts, and 18 female hypertrophied hearts. Values are mean ± SEM.

Functional parameters, except for heart rate in males, were reduced in all groups after ischemia as compared to corresponding pre-ischemic values. Post-ischemic function of hypertrophied hearts from both male and female rats was lower than corresponding non-hypertrophied hearts. Hypertrophied heart function after ischemia in females was lower than that in male hypertrophied hearts, except for post-ischemic coronary flow rates which did not differ between male and female hypertrophied hearts. All functional parameters in reperfused female non-hypertrophied hearts, except peak systolic pressure, were significantly lower than those in male non-hypertrophied hearts during reperfusion. The differences in post-ischemic function between male and female hearts remained when function was corrected for heart weight (Figure 1), except that function during reperfusion no longer differed significantly between male and female hypertrophied hearts. Compared to pre-ischemic values, recovery of function was lower in female hypertrophied (33.4 ± 4.1%) and non-hypertrophied (40.2 ± 4.3%) hearts than in corresponding male hypertrophied (48.4 ± 6.2%) and non-hypertrophied (64.4 ± 4.5%) hearts (p < 0.05). When external cardiac work is expressed relative to gender-matched non-hypertrophied hearts as a means to account for gender-related differences in function, post-ischemic functional recovery is comparably reduced in male (77.0 ± 9.5%) and female (83.0 ± 10.1%) hypertrophied hearts as compared to pre-ischemic values indicating that the impact of hypertrophy on post-ischemic recovery is similar in both genders.

### Myocardial substrate utilization

#### Glycolysis

**Figure 2:**
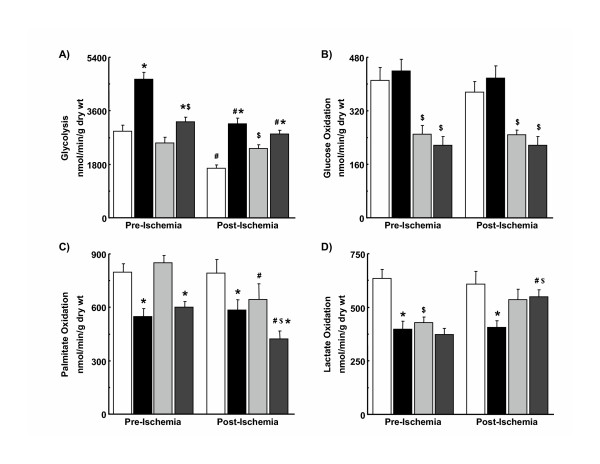
Glycolysis (A), glucose oxidation (B), palmitate oxidation (C), and lactate oxidation (D) in hypertrophied and non-hypertrophied hearts from male and female rats. White bar, male non-hypertrophied hearts; black bar, male hypertrophied hearts; light grey bar, female non-hypertrophied hearts; dark grey bar, female hypertrophied hearts. *, vs. sex-matched non-hypertrophied hearts at same time period (p < 0.05). #, vs. pre-ischemic value (p < 0.05). $, vs. corresponding male hearts at same time period (p < 0.05). Numbers per group: 12 to 16 male non-hypertrophied hearts, 8 to 14 male hypertrophied hearts, and 6 to 8 female non-hypertrophied hearts, 6 to 8 female hypertrophied hearts. Values are mean ± SEM.

Prior to ischemia, glycolysis was higher in hypertrophied hearts than in non-hypertrophied hearts from male and female rats (Figure 2A). Rates of glycolysis were greater in hypertrophied hearts from males than from females, while differences in glycolysis were not observed between male and female non-hypertrophied hearts. After ischemia, rates of glycolysis were significantly lower than pre-ischemic values in all groups except non-hypertrophied hearts from female rats. Glycolysis was, therefore, higher in female non-hypertrophied hearts than in male non-hypertrophied hearts. Rates of glycolysis continued to be accelerated in hypertrophied hearts after ischemia in males and females with rates in male and female hypertrophied hearts no longer significantly different.

### Glucose oxidation

Before ischemia, glucose oxidation was higher in male non-hypertrophied and hypertrophied hearts than in corresponding hearts from female rats (Figure 2B). Rates in hypertrophied hearts did not differ from those in non-hypertrophied hearts in either sex. Glucose oxidation after ischemia was not different from rates before ischemia in any group.

### Palmitate oxidation

Prior to ischemia, rates of palmitate oxidation were approximately 30% lower in hypertrophied hearts than in non-hypertrophied hearts from both male and female rats (Figure 2C). Sex of the animal did not influence palmitate oxidation rates in either non-hypertrophied or hypertrophied hearts before ischemia. In males, post-ischemic rates of palmitate oxidation in hypertrophied and non-hypertrophied hearts did not differ from those before ischemia. In contrast, palmitate oxidation decreased in hearts from female rats after ischemia compared to pre-ischemic values such that palmitate oxidation was significantly lower in hypertrophied hearts from females than in male hypertrophied hearts.

### Lactate oxidation

Compared to corresponding non-hypertrophied hearts, lactate oxidation before ischemia was lower in male hypertrophied hearts but was not different in female hypertrophied hearts (Figure 2D). Rates of lactate oxidation were higher in male than in female non-hypertrophied hearts. Lactate oxidation, therefore, did not differ in hypertrophied hearts from male and female rats. In male hypertrophied hearts, lactate oxidation after ischemia did not differ from that before ischemia. Lactate oxidation after ischemia increased in female hearts as compared to pre-ischemic values, achieving significance only in hypertrophied hearts. Post-ischemic rates of lactate oxidation in female hypertrophied hearts were now higher than those in hypertrophied hearts from male rats.

### Non-oxidative glycolysis

**Figure 3:**
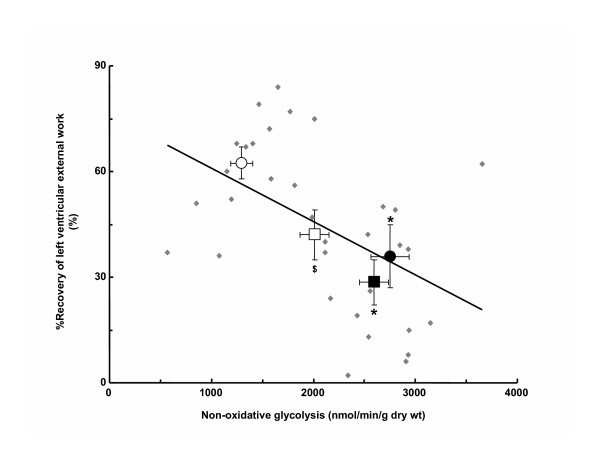
Relationship of post-ischemic contractile function to rates of non-oxidative glycolysis in hypertrophied and non-hypertrophied hearts from male and female rats. Percent (%) Recovery of left ventricular external work was calculated as the quotient of post-ischemic and pre-ischemic hydraulic power multiplied by 100. Non-oxidative glycolysis was calculated as the difference between rates of glycolysis and glucose oxidation. Regression analysis was performed using data from individual hearts (N = 33 hearts; R = -0.49, p < 0.05) and is expressed as mean data per group (N = 5 to 12 per group). Values are mean ± SEM. Open circle, Male Control. Closed circle, Male hypertrophy. Open square, Female Control. Closed square, Female Hypertrophy.

Prior to ischemia, non-oxidative glycolysis was higher in hypertrophied hearts than corresponding non-hypertrophied hearts in both sexes with rates in hypertrophied hearts from males greater than those from females (Table 3). Rates did not differ between male and female non-hypertrophied hearts. Non-oxidative glycolysis was higher in hypertrophied hearts than non-hypertrophied hearts but rates no longer differed between male and female hypertrophied hearts after ischemia. On the other hand, rates of post-ischemic non-oxidative glycolysis were significantly higher in non-hypertrophied hearts from females than from males. Of importance, recovery of heart function after ischemia showed a significant inverse relationship with rates of non-oxidative glycolysis (Figure 3).

**Table 3:** Non-oxidative glycolysis in hearts from sham-operated and aortic-constricted rats

	Male		Female	
		
	Control	Hypertrophy	Control	Hypertrophy
Pre-ischemic	2504 ± 180	4224 ± 231*	2274 ± 167	3017 ± 152*$
Post-ischemic	1259 ± 109#	2748 ± 187#*	2011 ± 144$	2595 ± 144#*

Values are Mean ± SEM. Sham-operated rats, Control. Aortic-constricted rats, Hypertrophy. Numbers are 5–18 per group; * vs. Sex-matched Control, p < 0.05; $ vs. corresponding Male heart at the same time period, p < 0.05; # vs. pre-ischemic value, p < 0.05.

### Myocardial glycogen

Content of glycogen did not differ between non-hypertrophied and hypertrophied hearts at the end of reperfusion in either males (136 ± 10 vs. 152 ± 9 μmol/g dry wt, p = NS) or females (118 ± 12 vs. 110 ± 8 μmol/g dry wt, p = NS). Myocardial glycogen was lower in female hearts than in male hearts, but was significantly lower only in female hypertrophied hearts as compared to male hypertrophied hearts (p < 0.05).

### Myocardial content and activity of selected metabolic proteins and enzymes

**Figure 4:**
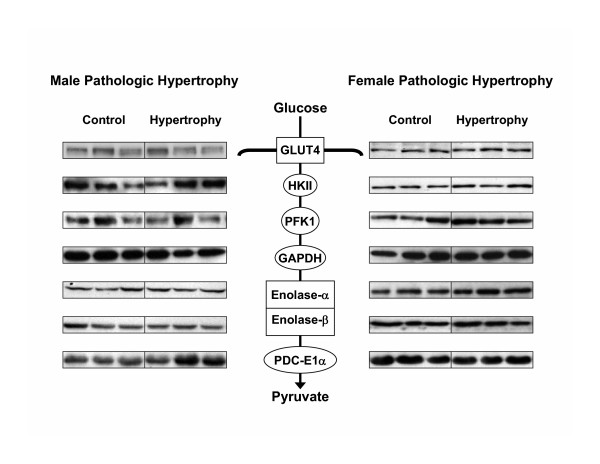
Representative immunoblots of key myocardial enzymes and proteins involved in glucose metabolism in non-hypertrophied (Control) and hypertrophied (Hypertrophy) hearts from male (Male Pathologic Hypertrophy) and female (Female Pathologic Hypertrophy) hearts. Each lane represents a single heart. GLUT-4, glucose transport protein-4; HKII, hexokinase-II; PFK-1, phosphofructokinase-1; GAPDH, glyceraldehyde-3-phosphate dehydrogenase; PDC E1α, pyruvate dehydrogeanse complex E1α subunit.

**Figure 5:**
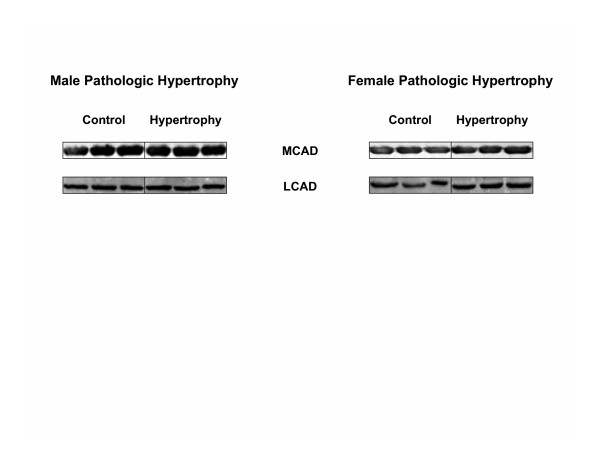
Representative immunoblots of myocardial enzymes and proteins involved in fatty acid oxidation in non-hypertrophied (Control) and hypertrophied (Hypertrophy) hearts from male (Male Pathologic Hypertrophy) and female (Female Pathologic Hypertrophy) hearts. Each lane represents a different heart. MCAD, medium chain acyl-CoA dehydrogenase; LCAD, long-chain acyl-CoA dehydrogenase.

**Figure 6:**
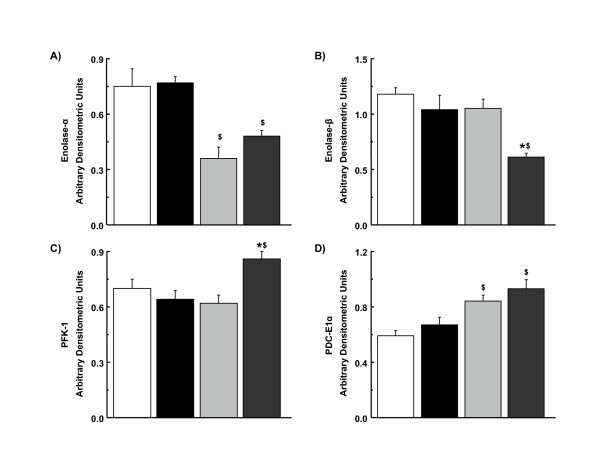
Densitometric analysis of selected myocardial enzymes and proteins involved in myocardial glucose metabolism in non-hypertrophied and hypertrophied hearts from male and female rats. (A) Enolase-α, (B) Enolase-β, (C) phosphofructokinase-1, and (D) pyruvate dehydrogeanse complex E1α subunit. White bar, male non-hypertrophied hearts; black bar, male hypertrophied hearts; light grey bar, female non-hypertrophied hearts; dark grey bar, female hypertrophied hearts. *, vs. sex-matched non-hypertrophied hearts (p < 0.05). $, vs. corresponding male hearts (p < 0.05). Values are expressed in arbitrary densitometry units, normalized to corresponding GAPDH densitometry units obtained from the same immunoblot. Numbers per group: 12 to 16 male non-hypertrophied hearts, 8 to 14 male hypertrophied hearts, and 6 to 8 female non-hypertrophied hearts, 6 to 8 female hypertrophied hearts. Values are mean ± SEM.

Differences in expression of key enzymes and proteins involved in glucose and fatty acid metabolism were not observed between male hypertrophied and non-hypertrophied hearts (Figures 4 and 5). In contrast, expression of PFK-1 and enolase-β was increased and decreased, respectively, in female hypertrophied hearts compared to non-hypertrophied hearts from female rats (Figures 4 and 6). As in males, differences in expression of MCAD and LCAD were not observed between female hypertrophied and non-hypertrophied hearts (Figure 5). Only minor sex-related differences in myocardial expression of these proteins and enzymes were detected (Figure 6). Specifically, expression of enolase-α, relative to GAPDH, was higher, while that of PDC E1α was lower in male hearts than in female hearts, regardless of the presence or absence of hypertrophy. Activity of PDC was not significantly different between hypertrophied and non-hypertrophied hearts in males (16.10 ± 2.27 vs. 14.48 ± 0.28 nmol min^-1 ^mg protein^-1^) or females (12.60 ± 1.12 vs. 15.97 ± 5.08 nmol min^-1 ^mg protein^-1^). There were also no significant gender related differences in activity of PDC.

## Discussion

### Gender, hypertrophy, and heart function

In the current study, gender was not found to significantly influence post-ischemic recovery of function in pressure overload hypertrophied hearts. Differences in post-ischemic function occurred largely because female sex was associated with greater dysfunction after ischemia than male sex. Interestingly, when compared to sex-matched non-hypertrophied hearts, the degree of functional impairment in female hypertrophied hearts was comparable to that in male hypertrophied hearts (Table 2 and Figure 1), indicating that the detrimental effect of hypertrophy on function after acute ischemia is similar in females than in males.

Few experimental studies have specifically compared the functional outcome of non-hypertrophied hearts from males and females after ischemia and reperfusion. In keeping with our results, Glick et al [34] found that post-ischemic functional recovery of isolated working hearts from female rats is lower than that of hearts from male rats when hearts are perfused with a solution containing glucose and fatty acid, a heart preparation and a perfusate composition very similar to that used in the current investigation. Our finding of a reduced functional recovery in female hearts after ischemia is also consistent with clinical data showing that women are at greater risk than men of a poor outcome after myocardial ischemia or revascularization procedures [7,9-12].

Gender-related differences in function of hypertrophied and non-hypertrophied hearts prior to ischemia were also observed. Pre-ischemic function of hypertrophied hearts was better maintained in female rats than in male rats, a finding that is entirely consistent with data from both clinical [1,4,35] and experimental [1-3] studies showing that female gender beneficially influences the myocardial response to hemodynamic overload. That the relative increase in heart mass did not differ significantly between male and female aortic-constricted rats is also consistent with previous work in which heart mass was shown to be similar in males and females early in the progression of experimental hypertensive heart disease [2].

### Gender, hypertrophy, and myocardial substrate utilization

Gender-related differences in myocardial substrate utilization were observed in hypertrophied and non-hypertrophied hearts before and after ischemia (Figure 2). Glycolysis was accelerated in hypertrophied hearts, regardless of sex, although the degree of acceleration was less in females than in males (Figure 2A). This acceleration of glycolysis is consistent with previous data from hypertrophied male rat hearts *in vitro *[19,23] and from hypertrophied dog [36] and rat [37] hearts *in vivo*. In male hypertrophied hearts, enhanced glycolytic rates were not accompanied by changes in expression of key metabolic enzymes and proteins (Figure 4), a finding that contrasts with previous observations that activity of a number of glycolytic enzymes is enhanced in hearts exposed to a pressure overload, albeit in different species [38,39]. Female hypertrophied hearts, on the other hand, demonstrated elevation of PFK-1, an enzyme having significant control strength for glycolytic flux, but a reduction in expression of another glycolyitc enzyme, enolase-β (Figures 4 and 6), the latter finding having been described previously in hypertrophied hearts from female rats [40].

In general, oxidation of glucose (Figure 2B) and, to a lesser extent, lactate (Figure 2D) was lower in female hearts than in male hearts, except after ischemia, where lactate oxidation rates in female hypertrophied hearts were higher than in corresponding male hearts. As with glycolysis, there was poor correspondence between measured rates of flux through the PDC, an enzyme that contributes significantly to the control of myocardial glucose and lactate oxidation, with its expression (Figure 4) and activity. That the patterns of use differ between glucose and lactate likely reflects the potential for fates of pyruvate derived from glucose and lactate to differ [41].

Myocardial glycogen did not differ between hypertrophied and non-hypertrophied hearts of either sex at the end of reperfusion, a finding in keeping with previous data obtained in male rats [19]. That glycogen content was lower in female hearts compared to male hearts may be a reflection of differences in the dynamic response of myocardial glycogen between males and females previously reported [42]. As glycogen is known to contribute significantly to energy production in male non-hypertrophied and hypertrophied hearts [28,43], gender-related differences in glycogen metabolism could have contributed to the functional differences observed in the current study.

Fatty acid oxidation was depressed in male and female hypertrophied hearts compared to corresponding non-hypertrophied hearts (Figure 2C), a finding consistent with previous results in hypertrophied hearts from male rats [23,44,45]. Despite reductions in measured rates of fatty acid oxidation, expression of MCAD and LCAD (Figure 5), key enzymes in the mitochondrial β-oxidation spiral, was not significantly altered in this model of mild compensated cardiac hypertrophy. The fall in fatty acid oxidation rates in female hearts after ischemia may be related to lower contractile function, as the differences disappear when fatty acid oxidation rates are normalized to work performed by the heart (data not shown). Notably, the pattern of use of the other myocardial substrates is not altered significantly when cardiac workload is accounted for. Taken together, hypertrophy-associated changes in substrate utilization rates and differences in substrate utilization between male and female hypertrophied hearts are not adequately accounted for by alterations in expression of metabolic enzymes and proteins, indicating that other factors, such as allosteric and/or covalent modification, translocation of glucose, fatty acid, or lactate transporters, substrate content, and product inhibition, are likely responsible.

### Post-ischemic heart function and myocardial substrate utilization

There is good evidence from experimental and clinical studies to indicate that the catabolic fate of glucose, and in particular the relative extent to which glucose is catabolized oxidatively as compared to non-oxidatively, is an important determinant of post-ischemic myocardial function in non-hypertrophied and hypertrophied hearts [19,20,22]. Substantial differences in non-oxidative glycolysis were observed among groups in the current study (Table 3). These differences were the result of gender-specific alterations in glucose catabolism with differences in the response of glycolysis to ischemia-reperfusion between male and female hearts being especially important because glucose oxidation rates were not similarly affected (Figure 2A and Table 3). Besides calculation from rates of glycolysis and glucose oxidation, non-oxidative glycolysis can also be determined from release of lactate and pyruvate by the heart, a parameter not measured in the current experiments. Results from previous studies indicate that an excellent correspondence exists between these two parameters [46].

When present, differences in non-oxidative glycolysis were accompanied by differences in post-ischemic function (Table 3 and Figure 1). In fact, we found a significant inverse relationship between non-oxidative glycolysis and recovery of contractile function after ischemia (Figure 3). The mechanism(s) by which non-oxidative glycolysis influences contractile function during reperfusion is not yet fully known. However, based upon the different proton stoichiometry of non-oxidative glucose catabolism as compared to glucose oxidation, which indicates 2 moles of protons are produced per mole of glucose catabolized non-oxidatively when the ATP formed is hydrolyzed [47,48], one possibility is that it is related to differences in net proton (H^+^) production arising from glucose catabolism [20]. Accelerated rates of H^+ ^production have been proposed to lead to increased calcium (Ca^2+^) overload as a result of successive trans-sarcolemmal H^+^/sodium (Na^+^) and Na^+^/Ca^2+ ^exchange, which in turn increases the energetic cost associated with the maintenance of ion homeostasis and, in doing so, leads to reduced post-ischemic contractile function and efficiency [20,49]. Future studies, in which pH and concentration of Na^+ ^and Ca^2+ ^in the myocardium of male and female hypertrophied and non-hypertrophied hearts are directly measured, will be required to determine if this is the case.

### Other potential mechanisms

In addition to variability in non-oxidative glycolysis, other factors likely contribute to the differences in post-ischemic contractile function observed. Following ischemia, coronary flow was significantly lower in non-hypertrophied hearts from female rats than in corresponding hearts from male rats (Table 2), a finding that may account in part for the poor recovery of female compared to male non-hypertrophied hearts. Lower rates of coronary flow in hypertrophied hearts than in non-hypertrophied hearts in both sexes may contribute to the reduced function in these hearts compared to corresponding non-hypertrophied hearts. Additionally, the genomic response to pressure overload is known to differ between hearts from males and females [50] with differential expression of a number of proteins relevant to functional outcome after ischemia, such as heat shock protein isoforms [51], ATP sensitive potassium channels [52], and sarcoplasmic reticulum calcium ATPase [52], having been described. The extent to which differences in expression of such proteins contribute to the post-ischemic outcomes of male and female hypertrophied hearts remains to be determined.

### Methodological considerations

The catabolism of endogenous substrates, such as glycogen and triglyceride, was not assessed in the current study. Given that endogenous substrates make significant contributions to overall energy production in the heart and the potential for gender-related differences to exist, it will be important to directly measure catabolism of endogenous substrates in future studies to determine their potential contribution to the functional outcomes observed. In the present study, contractile function of isolated working hearts was assessed at a single preload and afterload setting. A more comprehensive functional analysis using varying preload and afterload settings may have produced different results in male and female hypertrophied and non-hypertrophied hearts. Similarly, exposure to low-flow ischemia in contrast to no-flow ischemia may also have resulted in different post-ischemic outcomes than those in the current study. Future experiments will be required to clarify these important issues.

## Conclusion

Gender does not significantly influence post-ischemic function of hypertrophied hearts, even though female sex is detrimental to post-ischemic function of non-hypertrophied hearts. Differences in glucose catabolism may contribute to hypertrophy-induced and gender-related differences in post-ischemic function.

## Abbreviations

KH, Krebs-Henseleit; GLUT-4, glucose transporter-4; HK, hexokinase; GAPDH, glyceraldehyde-3-phosphate dehydrogenase; PDC, pyruvate dehydrogenase complex; PFK, phosphofructokinase; MCAD & LCAD, medium-chain and long-chain acyl-CoA dehydrogenases; ECL, enhanced chemiluminescent; ANOVA, analysis of variance; NMR, nuclear magnetic resonance.

## Competing interests

The author(s) declare that they have no competing interests.

## Authors' contributions

RS participated in writing the manuscript. RBW and HSL perfused the hearts and measured myocardial substrate utilization. HP and CA performed the immunoassays. AK and GAD and KMP provided antibodies and critically reviewed the manuscript. MFA is the senior author. All authors read and approved the final manuscript.

## Pre-publication history

The pre-publication history for this paper can be accessed here:


